# The role of lipids in mediating the effects of immune cells on Alzheimer’s disease risk: A network Mendelian randomization study

**DOI:** 10.1016/j.tjpad.2026.100509

**Published:** 2026-02-20

**Authors:** Xinyu Yang, Jingjing Jiang, Wenjing Li, Rui Pan, Yanjie Li

**Affiliations:** a Medical College of Rehabilitation, Henan University of Chinese Medicine, Zhengzhou 450046, China; b Department of Rehabilitation, Henan Provincial Hospital of Traditional Chinese Medicine, Zhengzhou 450002, China

**Keywords:** Alzheimer's disease, Immune cells, Lipids, Mediation analysis, Mendelian randomization

## Abstract

**Background:**

Observational studies have shown associations between immune cells, lipids, and Alzheimer’s disease (AD), but their specific causal relationships and the mediating role of lipids remain unclear.

**Methods:**

Within a network Mendelian randomization (MR) framework, we first applied two-sample univariable MR to assess the causal effects of immune cells and lipids on AD. Then, multivariable MR was used in mediation analyses to determine whether lipids mediate the effects of immune cells on AD. Finally, reverse MR analyses were performed to minimize potential bias from reverse causation. The inverse variance weighted method was used as the primary estimator.

**Results:**

The analysis revealed that elevated levels of CD33 on CD33dim HLA DR+ CD11b+ and CD33 on CD33dim HLA DR+ CD11b- were associated with an increased risk of AD. Mediation analysis further indicated that polyunsaturated fatty acids are protective lipid metabolites for AD and partially mediate the effects of the aforementioned immune cells on AD, with mediation proportions of 3.70 % and 3.67 %, respectively.

**Conclusion:**

This study provides new insights into how immune cells may influence AD pathogenesis through lipid metabolism. It also offers a theoretical basis and potential direction for developing immune–lipid-based strategies for AD prevention and intervention.

## 1 Introduction

Alzheimer’s disease (AD) is a neurodegenerative disorder characterized by impairments in multiple cognitive domains, including memory, language, and reasoning, which severely affect patients’ independence in daily activities [1]. Epidemiological studies show that more than 55 million people worldwide live with dementia, and this number is projected to rise to 152.8 million by 2050 [2]. According to the 2024 Alzheimer’s Disease Facts and Figures report, AD has become the fifth leading cause of death among individuals aged 65 and older in the United States, imposing a substantial medical and economic burden on families and society [3]. Although studies have identified β-amyloid (Aβ) deposition and abnormally phosphorylated tau protein as the primary pathological hallmarks of AD, current clinical interventions remain limited in slowing disease progression and improving outcomes [4,5]. Therefore, a deeper understanding of the pathophysiological mechanisms of AD and the exploration of novel therapeutic strategies are urgently needed.

In recent years, studies have revealed that immune regulation plays a crucial role in the pathogenesis of AD [6]. Microglia are the principal immune cells within the central nervous system. During the early stages of AD, Aβ deposition can trigger microglial activation, enabling the recognition and clearance of Aβ and other abnormal substances [7]. However, excessive or sustained activation of microglia leads to the release of pro-inflammatory cytokines, triggering neuroinflammatory responses and accelerating AD progression [8]. In addition, the peripheral immune system has also been shown to participate in immune regulation during AD. For instance, compared with healthy controls, AD patients exhibit a significant increase in CD8+ *T* cells within the perivascular spaces of the hippocampus, accompanied by Aβ deposition [9]. Furthermore, Van Olst et al. [10] demonstrated a strong association between alterations in peripheral immune status and the clinical stages of AD, from early to late phases. Collectively, these findings suggest that immune cells may play an important regulatory role in the pathogenesis of AD.

Lipids are essential biomolecules that maintain cellular structure and physiological function, playing critical roles in energy metabolism, signal transduction, and the regulation of cellular homeostasis [11]. Previous studies have found that dysregulated lipid metabolism is closely associated with Aβ formation and tau pathology, and the disruption of lipid homeostasis accelerates the progression of AD [12]. Moreover, immune regulatory processes can further contribute to AD pathology by affecting lipid metabolism [13]. For example, loss of function of the Triggering Receptor Expressed on Myeloid Cells 2 (TREM2) on microglia results in lipid metabolic abnormalities, which in turn exacerbate Aβ deposition and elevate AD risk [14]. Although previous studies have identified associations among immune cells, lipids, and AD, the diversity of immune cells and lipids and the complexity of their interactions mean that whether there are causal relationships among them has not been elucidated. Furthermore, these findings are prone to confounding and reverse causation bias. Therefore, their effects on AD require further investigation.

Mendelian randomization (MR) is a method that uses genetic variants as instrumental variables (IVs) to explore causal relationships between exposures and outcomes [15]. Because the distribution of genetic variants is generally determined at conception and not influenced by environmental or lifestyle factors, MR can effectively minimize confounding and reverse causation compared with traditional observational studies [16]. In this study, we conducted two-sample MR and mediation analyses using the most comprehensive summary-level genome-wide association study (GWAS) data available for immune cells, lipids, and AD. The aim was to elucidate their potential contributions to the pathophysiological mechanisms of AD and to provide novel perspectives and theoretical foundations for disease prevention and intervention.

## 2 Methods and materials

### 2.1 Study design

**Fig. 1:**
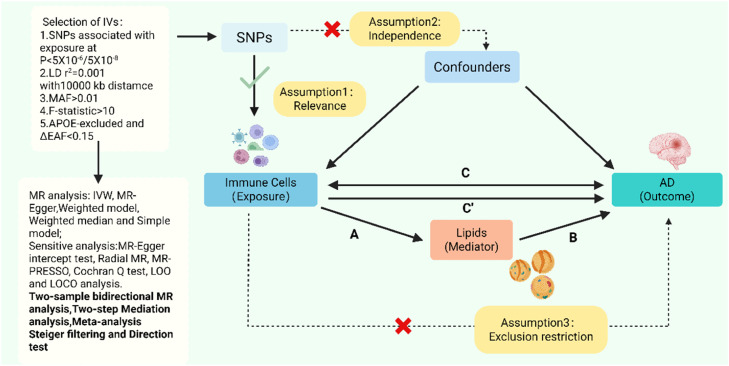
Overview and research content of Mendelian randomization.

As shown in Fig. 1, the network MR analysis in this study consisted of two main parts. The first part involved assessing the causal relationships between 731 immune cell traits and AD (Step C). These results were further validated using replication cohorts and meta-analysis. The second part examined lipid-mediated pathways linking immune cells to AD (Steps A, B, and C’). In addition, to minimize the influence of reverse causation, we not only performed Steiger filtering and directionality tests but also systematically explored the reverse causal model from AD to lipids to immune cells. To ensure result reliability, the genetic variants selected as IVs in MR analysis must satisfy three key assumptions: (1) Relevance — the IVs are strongly associated with the exposure; (2) Independence — the IVs are independent of confounders; and (3) Exclusion restriction — the IVs influence the outcome only through the exposure [17]. All data used in this study were obtained from publicly available GWAS, and thus no additional ethical approval was required. This study adheres to the STROBE-MR reporting guidelines [18].

### 2.2 Data sources

Summary statistics for immune cell phenotypes were obtained from the GWAS Catalog (GCST90001391–GCST90002121), based on a study of 3757 individuals of European ancestry, encompassing a total of 731 immune traits [19]. AD data were derived from the phase I summary statistics of the European Alzheimer & Dementia Biobank (EADB), comprising 85,934 cases and 401,577 controls of European ancestry [20]. Additionally, replication cohort data for AD were obtained from the FinnGen consortium (finngen_R12_G6_ALZHEIMER), including 13,964 CE cases and 486,384 controls [21]. The lipid phenotype data were obtained from a study involving 33 cohorts with a total of 136,016 participants, encompassing 213 lipid traits [22]. Detailed information on the summary statistics used in this study is provided in Supplementary Table S1.

### 2.3 Selection of instrumental variables

In this study, single nucleotide polymorphisms (SNPs) were defined as IVs. To ensure the reliability and accuracy of MR results, several rigorous quality control measures were implemented. First, we set the significance threshold at *P* < 5 × 10⁻⁸. However, under this stringent threshold, the number of IVs for immune cell phenotypes was limited, which was insufficient for subsequent analyses. Therefore, we relaxed the threshold for immune phenotypes to *P* < 5 × 10⁻⁶, while maintaining *P* < 5 × 10⁻⁸ for lipid traits and AD. Second, linkage disequilibrium (LD) clumping was performed using r² < 0.001 and a clumping window of 10,000 kb to ensure minimal overlap and independence among SNPs. Next, the F-statistic for each SNP was calculated using the formulas R² = 2 × EAF × (1-EAF) × β² / [2 × EAF × (1-EAF) × β² + 2 × EAF × (1-EAF) × *N* × SE²] and *F* = *R*² × (N-2)/(1-R²), with SNPs having *F* < 10 considered weak instruments. The minor allele frequency (MAF) was derived from the effect allele frequency (EAF) to eliminate the potential bias introduced by rare variants [23]. Based on these calculations, only SNPs with *F* > 10 and MAF > 0.01 were retained as valid IVs [16]. Additionally, trait-level R² and F-statistics were computed to assess the overall IV strength, with trait-level *F* > 10 considered indicative of sufficient instrument strength.

Among the known genetic risk factors for AD, the apolipoprotein E (APOE) gene has the strongest and most widespread effects, playing a significant regulatory role in circulating lipid levels [24]. Therefore, to prevent bias in causal inference from genetic variation within the APOE region, all SNPs located in the APOE gene region (hg19: chr19: 44.4–46.5 Mb) were excluded during the selection of immune phenotype SNPs. Given that the immune cell GWAS data were derived from the genetically isolated SardiNIA cohort, to ensure the portability of SNP–exposure associations to broader European populations, we compared each SNP’s EAF in the SardiNIA dataset with that in the European reference panel from the 1000 Genomes Phase 3 (1000 G P3 EUR panel). SNPs with an absolute EAF difference greater than 0.15 were excluded [25]. Similarly, to minimize ancestry-related bias, the same EAF comparison strategy was applied during the selection of lipid-associated SNPs. In addition, to further mitigate horizontal pleiotropy, we used the PheWeb and GWAS Catalog databases to examine whether SNPs associated with positive traits were also linked to other phenotypes. When an SNP showed suggestive associations (*P* < 1 × 10⁻⁵) with unrelated traits in both databases, it was considered potentially pleiotropic and removed, followed by re-performing the MR analysis. Finally, during the harmonization process, palindromic SNPs with ambiguous alleles were removed to prevent bias due to strand orientation or allele coding errors (Supplementary Table S6).

### 2.4 Primary Mendelian randomization analysis

To estimate the causal relationship between exposures and outcomes, we conducted two-sample MR analyses, in which β coefficients were used to quantify the causal effects of exposures on outcomes. Five commonly used MR methods were applied: inverse-variance weighted (IVW), weighted median, MR-Egger regression, simple mode, and weighted mode. IVW was used as the primary analytical method, while the other four served as complementary approaches. The IVW method assumes that all genetic variants are valid instruments and estimates the causal effect as the slope of a weighted linear regression [26]. The weighted median approach can provide consistent estimates even when up to 50 % of the IVs are invalid [27]. MR-Egger regression can generate valid causal estimates even when all genetic variants exhibit pleiotropy [28]. The simple mode and weighted mode methods provide robust causal estimates as long as the majority (or the highest-weighted) IVs reflect the actual causal effect, even in the presence of invalid instruments [29]. Results were considered statistically significant only when the IVW p-value was <0.05 and the β directions from all five methods were consistent. In addition, to address the issue of multiple testing, we applied eigen-decomposition to estimate the effective number of independent tests and thereby obtain an appropriate multiple testing correction [30].

### 2.5 Sensitivity analyses

For sensitivity analyses, Cochran’s Q test and the MR-Egger intercept test were used to assess heterogeneity and horizontal pleiotropy, respectively. When *P* > 0.05, the results suggested no evidence of substantial heterogeneity or horizontal pleiotropy. The MR-PRESSO and Radial MR methods were applied to identify and remove outlier SNPs, yielding more accurate and reliable estimates of causal effects [31,32]. Leave-one-out (LOO) and leave-one-chromosome-out (LOCO) analyses were performed to evaluate the influence of individual IVs or single chromosomes on the overall causal estimates [33,34]. Considering that some IVs were shared among different immune phenotypes, we further removed SNPs used in two or more traits and repeated the MR analysis to assess their potential impact. Changes in effect direction or *a* ≥ 30 % difference in the IVW estimate after SNP removal were interpreted as evidence of sensitivity to cross-trait IV overlap.

### 2.6 Meta-analysis

For phenotypes that showed significant associations in the initial MR analysis, replication analyses were conducted using AD data from the FinnGen study. Subsequently, a meta-analysis was performed to combine the IVW results from both datasets to assess the reliability of the findings. The significance threshold for the meta-analysis was set at *P* < 0.05. Phenotypes validated through meta-analysis were subsequently included in the mediation analyses.

### 2.7 Mediation analysis

First, the inclusion criteria for candidate lipid mediators were as follows: (1) a causal association between the mediator and AD; (2) a causal relationship between immune cells and the mediator; (3) the mediator must exert a direct causal effect on AD independent of immune cells; and (4) the direction of the mediation effect must be consistent with the total effect. In the preceding analyses, we had already determined the total effect of immune cells on AD (βC). Therefore, the subsequent mediation analysis was conducted in the following steps: (1) Perform univariable MR (UVMR) analysis to identify lipid traits with causal associations with AD. (2) Conduct UVMR analysis to evaluate the causal effect of significant immune cell traits on the identified lipid mediators (βA). (3) Conduct multivariable MR (MVMR) analysis to assess the direct causal effect of lipids on AD (βB) after adjusting for immune cell effects. (4) The mediation effect was calculated as βA × βB; the direct effect as βC-(βA × βB); and the proportion mediated as (βA × βB)/βC. Delta and bootstrap methods were used to estimate the 95 % CIs of the mediation effect and the proportion mediated. This dual approach enables cross-validation of the precision and robustness of the estimates, particularly given the potential instability of proportion-mediated calculations when total effects are small. Furthermore, to ensure the comparability of effect estimates in the mediation analysis, SNP harmonization was performed between steps C′ and A, using the most consistent set of IVs possible.

### 2.8 MVMR residual diagnostics

To assess residual pleiotropy in the MVMR analysis, we first calculated the Sanderson–Windmeijer conditional F-statistic to evaluate the overall strength of the IVs and to exclude weak instrument bias. Next, the multivariable Q-statistic was applied to test for heterogeneity, and the intercept term from multivariable MR-Egger regression was used to detect horizontal pleiotropy, thereby ensuring the robustness and reliability of the causal estimates. In addition, to rigorously control for potential confounding by APOE, an APOE-excluded sensitivity analysis was performed: after removing the APOE genomic region, MVMR analysis was re-run. If the direction or magnitude of causal estimates remained consistent after APOE exclusion, the results were considered robust and independent of APOE-driven effects.

### 2.9 Reverse causality analysis

We first applied Steiger filtering to the selected SNPs. Then, we conducted the directionality test by comparing the variance explained by the IVs in the exposure versus the outcome to verify the correctness of the causal direction [35]. In addition, we conducted a complete reverse MR analysis along the AD–lipid–immune cell pathway to exclude potential reverse causality bias and strengthen the credibility of causal inference. In the reverse MR analysis, the Benjamini–Hochberg method was used to adjust the IVW results for the false discovery rate (FDR), with statistical significance defined as p-FDR < 0.05 [36].

All analyses were conducted using R software version 4.5.0. The "TwoSampleMR" (version 0.6.16), "MVMR" (version 0.4.2) and "MendelianRandomization" (version 0.10.0) packages were used for MR analyses. The "MRPRESSO" (version 1.0) and "RadialMR" (version 1.2) packages were used for sensitivity analyses. The "ggplot2″ (version 3.5.2), "forestploter" (version 3.1.7), and "ComplexHeatmap" (version 2.24.0) packages were used for data visualization.

## 3 Results

Selection of IVs

Among the 731 immune cell phenotypes, after excluding SNPs within the APOE region and removing variants with an EAF difference greater than 0.15 compared to the 1000 G P3 EUR reference panel, a total of 8391 SNPs were retained. The SNP-level F-statistics ranged from 20.871 to 3159.289, and the trait-level F-statistics ranged from 21.560 to 740.629. Similarly, following the same EAF filtering criteria, 12,518 SNPs were selected from the 213 lipid traits, with SNP-level F-statistics ranging from 29.717 to 5088.128 and trait-level F-statistics ranging from 47.439 to 350.780. For AD used as the exposure variable, 56 SNPs were extracted, with SNP-level F-statistics ranging from 30.384 to 519.110 and a trait-level F-statistic of 87.888. All SNPs and traits had F-statistics greater than 10 and MAF above 0.01, effectively excluding bias from weak instrumental variables (Supplementary Tables S2–S5).

### 3.1 Two-sample MR analysis of immune cells on AD

**Fig. 2:**
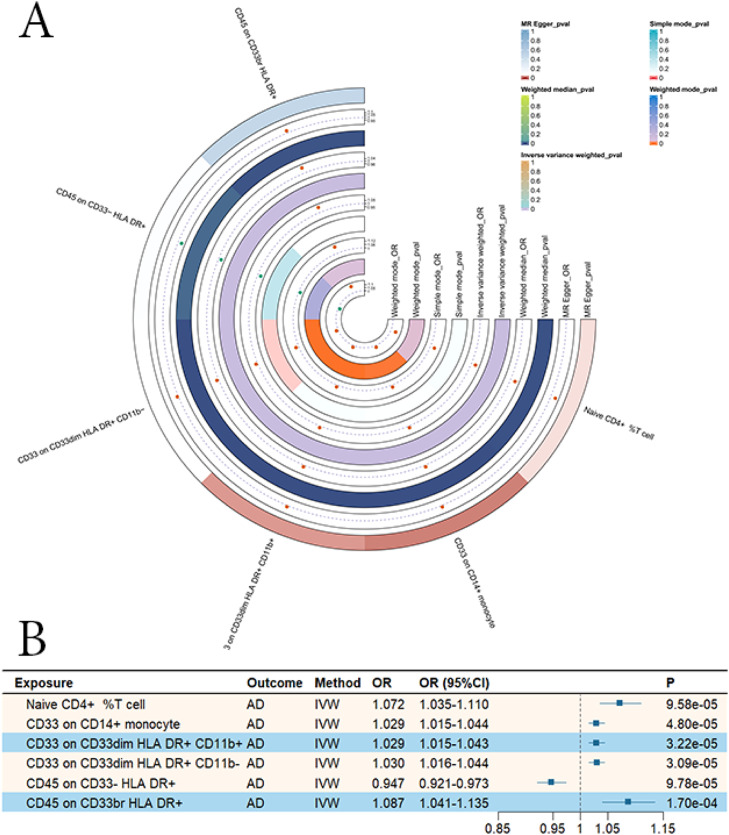
Mendelian randomization analysis of the causal relationship between immune cells and Alzheimer’s disease. A. Heatmap of MR analysis results. B. Forest plot of MR analysis results.

We first performed two-sample MR analyses to assess the causal effects of immune cell traits on AD. MR-PRESSO and Radial MR were used to identify and remove outlier SNPs. The PheWeb and GWAS Catalog databases were consulted to exclude potentially pleiotropic variants, ensuring the robustness of downstream analyses. The effective number of independent tests estimated by eigen-decomposition was 291; thus, the significance threshold for P-values was set at 1.72 × 10⁻⁴ (0.05/291). According to the IVW results, six immune phenotypes exhibited significant causal associations with AD. Specifically, higher levels of Naive CD4+ % T cells, CD33 on CD14+ monocytes, CD33 on CD33dim HLA DR+ CD11b+, CD33 on CD33dim HLA DR+ CD11b-, and CD45 on CD33br HLA DR+ were associated with increased AD risk, whereas CD45 on CD33- HLA DR+ was associated with reduced AD risk (Fig. 2; Supplementary Table S7). The MR estimates were directionally consistent across all analytical methods, and no evidence of pleiotropy or heterogeneity was detected in sensitivity analyses (Supplementary Table S8). The data visualizations of Radial MR, LOO, and LOCO analyses, as well as scatter plots, are provided in the Supplementary Materials.

Furthermore, to further investigate the specific causal effects of these immune phenotypes, we repeated MR analyses for the six immune traits after removing their shared instrumental variables. The IVW results indicated that the causal effect directions of all exposures remained consistent. However, the effect estimates for CD33 on CD14+ monocytes, CD45 on CD33- HLA DR+, and CD45 on CD33br HLA DR+ changed markedly (≥30 %) compared with the original analysis, suggesting that their causal inferences may be substantially influenced by shared genetic variants. In contrast, the effect estimates for Naive CD4+ % T cells, CD33 on CD33dim HLA DR+ CD11b+, and CD33 on CD33dim HLA DR+ CD11b- changed minimally (<30 %) after SNP exclusion, indicating that their causal effects were more likely driven by non-shared SNPs and thus more robust (Supplementary Table S9).

### 3.2 Meta-analysis

**Fig. 3:**
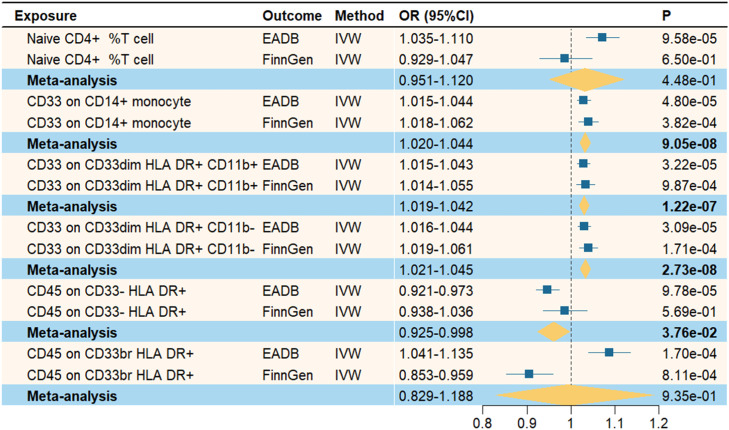
Meta-analysis of the causal relationship between immune cells and Alzheimer’s disease.

To enhance the stability of our findings, we repeated the MR analyses using AD datasets from different sources. Next, the two sets of IVW results were combined using meta-analysis. As shown in Fig. 3, the meta-analysis results for CD33 on CD14+ monocytes, CD33 on CD33dim HLA DR+ CD11b+, CD33 on CD33dim HLA DR+ CD11b-, and CD45 on CD33- HLA DR+ remained statistically significant (*P* < 0.05), with consistent effect directions compared with the initial MR analyses. Therefore, these four immune cell phenotypes were included in the subsequent mediation MR analysis. In contrast, Naive CD4+ % T cells and CD45 on CD33br HLA DR+ did not replicate in the meta-analysis (*P* > 0.05), suggesting potential heterogeneity or instability in their causal relationships with AD. These findings support the reliability and robustness of our MR study results.

### 3.3 Mediation analysis of lipids

**Fig. 4:**
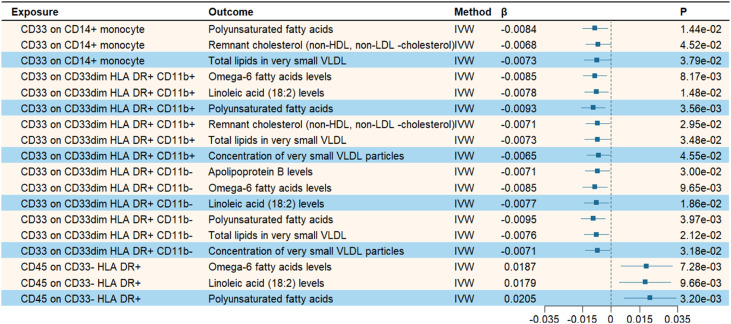
Mendelian randomization analysis of the causal relationship between immune cells and lipids.

In exploring potential mediators, we initially selected 213 lipid traits to examine their causal effects on AD. The effective number of independent tests estimated by eigen-decomposition was 50. After removing outliers using MR-PRESSO and Radial MR, the IVW analysis revealed that 42 lipid traits were significantly associated with AD risk (*P* < 0.001 = 0.05/50) (Supplementary Table S10). We then further investigated the causal relationships between four immune cell phenotypes (CD33 on CD14+ monocytes, CD33 on CD33dim HLA DR+ CD11b+, CD33 on CD33dim HLA DR+ CD11b-, and CD45 on CD33- HLA DR+) and the aforementioned lipid traits associated with AD. As shown in Fig. 4, when CD33 on CD14+ monocytes and CD45 on CD33- HLA DR+ were used as exposures, three lipids each were found to have causal relationships; CD33 on CD33dim HLA DR+ CD11b+ and CD33 on CD33dim HLA DR+ CD11b- were each significantly associated with six lipid traits (Supplementary Table S11). The MR estimates were directionally consistent across all analytical methods, and sensitivity analyses revealed no evidence of pleiotropy or heterogeneity (Supplementary Tables S13 and S14).

**Fig. 5:**
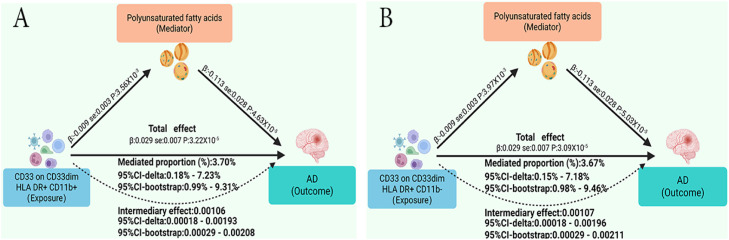
Mediating role of lipids in the effect of immune cells on Alzheimer’s disease.

Finally, after excluding potentially pleiotropic SNPs using the PheWeb and GWAS Catalog databases, we performed mediation MR analyses to evaluate whether the four immune cell traits influence AD through lipid mediators. We calculated the mediation effects and their proportions (Supplementary Table S12). The results indicated that elevated levels of CD33 on CD33dim HLA DR+ CD11b+ and CD33 on CD33dim HLA DR+ CD11b- could increase the risk of AD by reducing polyunsaturated fatty acid (PUFA) levels. As shown in Fig. 5, the mediation effect of PUFA on the pathway from CD33 on CD33dim HLA DR+ CD11b+ to AD was β = 0.00106 (95 % CI-delta: 0.00018–0.00193; 95 % CI-bootstrap: 0.00029–0.00208), with a mediation proportion of 3.70 % (95 % CI-delta: 0.18 %–7.23 %; 95 % CI-bootstrap: 0.99 %–9.31 %). For CD33 on CD33dim HLA DR+ CD11b-, the mediation effect was β = 0.00107 (95 % CI-delta: 0.00018–0.00196; 95 % CI-bootstrap: 0.00029–0.00211), and the mediation proportion was 3.67 % (95 % CI-delta: 0.15 %–7.18 %; 95 % CI-bootstrap: 0.98 %–9.46 %). In the sensitivity analyses, all Sanderson–Windmeijer conditional F-statistics were greater than 10, and no significant horizontal pleiotropy or heterogeneity was detected (Supplementary Table S15). Moreover, after repeating the MVMR analyses with the APOE region excluded, the results remained unchanged.

### 3.4 Reverse causal inference

To minimize the influence of reverse causation, Steiger filtering was applied to the SNPs used for immune cells and lipid traits, followed by directionality testing at the phenotype level. Both analyses supported the causal direction from immune cells and lipids to AD (Supplementary Tables S16 and S17). In addition, to further investigate the possibility of a reverse causal pathway (AD → lipids → immune cells), we conducted reverse MR analyses corresponding to the previous analyses (Supplementary Tables S18 and S19). The IVW results showed that, in the MR analysis of AD on 213 lipid traits, 100 lipids reached statistical significance (p-FDR < 0.05). However, in the MR analysis of AD on 731 immune cell traits, no immune phenotype showed a significant causal association with AD (p-FDR < 0.05). Therefore, there is insufficient evidence to support a reverse causal pathway in which AD drives lipid alterations that subsequently influence immune cell changes.

## 4 Discussion

This study represents the first MR analysis to investigate the causal relationship between immune cells and AD using lipids as mediating factors. After rigorous sensitivity analyses and validation through meta-analysis, we identified significant and robust causal effects of CD33 on CD33dim HLA DR+ CD11b+ and CD33 on CD33dim HLA DR+ CD11b- on AD risk. In contrast, the causal effects of CD33 on CD14+ monocytes, CD45 on CD33- HLA DR+, and CD45 on CD33br HLA DR+ on AD were likely influenced by shared genetic variants rather than reflecting specific immune effects. Moreover, Naive CD4+ % T cells and CD45 on CD33br HLA DR+, which showed significance in the initial MR analysis, did not replicate in the meta-analysis. This inconsistency may be attributed to heterogeneity between datasets or bias in effect estimation. The mediation analysis further identified PUFA as a protective lipid metabolite against AD. Elevated levels of CD33 on CD33dim HLA DR+ CD11b+ and CD33 on CD33dim HLA DR+ CD11b- increased AD risk through the reduction of PUFA levels, with mediation proportions of 3.70 % and 3.67 %, respectively. These findings suggest that part of the immune-driven risk for AD may be mediated through lipid metabolic pathways. Overall, this study provides genetic evidence for a causal chain linking immunity, lipid metabolism, and AD. It also suggests that lipid metabolism may represent a critical entry point for understanding immune-mediated AD pathology and for developing future therapeutic strategies.

CD33 on CD33dim HLA DR+ CD11b+ and CD33 on CD33dim HLA DR+ CD11b- belong to the myeloid cell lineage, which mainly comprises monocytes, dendritic cells, tissue macrophages, and granulocytes, playing key roles in antigen presentation, phagocytic clearance, and inflammatory responses [37]. CD33 is a member of the sialic acid–binding immunoglobulin-like lectin (Siglec) family and exerts immunosuppressive regulatory functions [38]. Genetically, multiple GWAS studies have identified CD33 variants (e.g., rs3865444, rs12459419) associated with AD susceptibility, and elevated CD33 expression has been linked to increased AD risk [39,40]. Furthermore, previous studies have observed that CD33 mRNA levels are significantly upregulated in both the brain and peripheral blood of AD patients [41,42]. Moreover, Griciuc et al. [43] found in APP/PS1 transgenic mice that early suppression of CD33 expression reduced pro-inflammatory gene levels, decreased insoluble Aβ42 deposition, and alleviated amyloid plaque burden in the cortex and hippocampus. Collectively, these findings suggest that CD33 may play a crucial regulatory role in the onset and progression of AD.

HLA-DR is a major histocompatibility complex (MHC) class II molecule expressed on the surface of immune-active cells, primarily involved in antigen presentation within the immune system [44]. Genetically, Steele et al. [45] found that the MHC class II region containing the HLA-DR gene was significantly associated with AD risk and correlated with accelerated cognitive decline and elevated inflammatory markers. Moreover, compared with normal controls, abundant HLA DR+ activated microglia were observed in the hippocampus of AD patients, clustering around amyloid plaques, neurofibrillary tangles, and areas of neuronal degeneration [46]. Furthermore, Mancuso et al. [47] observed in a humanized Aβ mouse model that HLA-DR–high immune cell subsets were markedly enriched around Aβ plaques and increased progressively with disease advancement. Therefore, these findings suggest that HLA DR+ expression is closely associated with the persistent neuropathological activity observed in AD.

In addition to immune cells, lipid metabolism also plays a crucial role in the pathogenesis of AD [12]. PUFA is classified based on the position of the first double bond in its carbon chain into ω−3 PUFA (N3FA) and ω−6 PUFA (N6FA), such as docosahexaenoic acid (DHA) and γ-linolenic acid. PUFA in the brain is mainly derived from the peripheral circulation. It is synthesized in the liver, transported in the form of lipoproteins, and crosses the blood–brain barrier to enter neurons, where it maintains essential functions such as neuronal signaling, neurogenesis, and the regulation of inflammation [48]. For instance, DHA enhances synaptogenesis and improves long-term memory function by upregulating the expression of postsynaptic density protein 95 (PSD95) [49]. Moreover, several studies have demonstrated that long-term DHA supplementation improves cognitive performance or delays cognitive decline in older adults and individuals with mild cognitive impairment [50,51]. In addition, PUFA also plays an important role in the onset and progression of AD. A prospective cohort study found that higher blood levels of N3FA, N6FA, and total PUFA were associated with a lower risk of dementia incidence and mortality [52]. This may be attributed to PUFA, which exerts neuroprotective effects through multiple pathways, including the inhibition of Aβ production, downregulation of pro-inflammatory cytokine expression, attenuation of neuroinflammation, and promotion of anti-apoptotic signaling, thereby reducing neuronal damage [53,54].

However, there is currently no direct evidence demonstrating that CD33 on CD33dim HLA DR+ CD11b+ and CD33 on CD33dim HLA DR+ CD11b- can directly regulate PUFA levels. Previous studies have shown that under antigenic stimulation, the expression of cyclooxygenase-2 and 5-lipoxygenase is significantly upregulated in HLA DR+ monocytes, thereby promoting the metabolism of arachidonic acid (a type of N6FA) to generate proinflammatory mediators such as prostaglandin E₂ and leukotriene B₄, which in turn mediate immune-inflammatory responses [55]. These findings suggest that elevated levels of CD33 on CD33dim HLA DR+ CD11b+ and CD33 on CD33dim HLA DR+ CD11b- may reduce PUFA levels, disrupt lipid homeostasis, and exacerbate neuroinflammation, ultimately increasing the risk of AD. It will be essential in future work to employ multi-omics analyses and functional assays to confirm this immune–lipid interplay and clarify its mechanistic link to AD progression.

The strength of this study lies in its use of the most up-to-date and comprehensive GWAS datasets to explore the causal relationship between immune cells and AD. Secondly, rigorous selection criteria and multiple sensitivity analyses were applied to ensure the reliability of the MR results. Furthermore, replication analyses and meta-analytic validation further reinforced the findings and confirmed the causal effects. In the mediation analysis, we investigated possible mediators through which immune cells may influence AD. Finally, reverse MR analyses were conducted to explicitly examine the AD–lipid–immune cell causal direction, thereby minimizing potential reverse causality bias.

Nevertheless, this study has several limitations. First, the immune cell data were derived from the genetically isolated Sardinian population. Although sensitivity analyses were performed to control for potential bias, future studies using broader European cohorts and stricter selection thresholds are needed to verify the generalizability of the results. Second, the AD GWAS datasets from EADB and FinnGen differ in recruitment strategies, age distributions, and case definitions, which may introduce survival or selection bias. Moreover, causal effects in this study were reported as odds ratios (OR), which are “non-collapsible” effect measures—meaning their magnitudes may be influenced not only by the true causal effect but also by outcome prevalence and covariate distribution within the sample. Notably, this non-collapsibility affects only the magnitude of the effect estimates but not the causal direction. Therefore, our inference regarding the direction of immune or lipid effects on AD risk remains valid. Future studies using individual-level data or liability-scale adjusted summary statistics may provide more precise quantitative interpretations. Third, although the lipid GWAS used in this study was predominantly based on European populations, it included a small proportion of non-European participants. To reduce potential ancestry-related bias, we performed corresponding sensitivity analyses; however, further validation using GWAS data from more genetically homogeneous populations is still required. Fourth, although the delta and bootstrap methods were used to estimate mediation effects and proportions with 95 % CIs, the use of summary-level data precluded testing for potential nonlinear relationships or exposure–mediator interactions, which may limit full representation of biological complexity. Fifth, although strict selection criteria and multiple sensitivity analyses were implemented, residual or undetected pleiotropy cannot be entirely ruled out. Future studies integrating more comprehensive SNP functional annotations and broader lipid-category MVMR models are needed to further validate the robustness of the results. Finally, future studies with larger sample sizes, broader population coverage, and experimental validation are required to comprehensively elucidate the causal mechanisms linking immune cells, lipids, and AD.

## 5 Conclusion

The MR analysis in this study revealed causal effects of CD33 on CD33dim HLA DR+ CD11b+ and CD33 on CD33dim HLA DR+ CD11b- on AD. Further mediation analyses suggested that PUFA may partially mediate this causal pathway. These findings provide new insights into how immune cells may influence AD pathogenesis through lipid metabolism. It also offers a theoretical basis and potential direction for developing immune–lipid-based strategies for AD prevention and intervention.

## Funding

This work was supported by the National Administration of Traditional Chinese Medicine Science and Technology Project "Zhang Zhongjing Inheritance and Innovation Special Project" (GZY-KJS-2022–043–3) and Traditional Chinese Medicine Discipline Project of Henan Province's "Double First Class" Creation Project (HSRP-DFCTCM-2023–3-11).

## Declaration

### Ethics approval and consent to participate

Not applicable.

### Consent for publication

Not applicable.

### Generative AI in scientific writing

This study did not use generative AI or AI-assisted technologies.

## CRediT authorship contribution statement

**Xinyu Yang:** Writing – review & editing, Writing – original draft, Software, Conceptualization. **Jingjing Jiang:** Writing – review & editing, Validation. **Wenjing Li:** Resources, Data curation. **Rui Pan:** Data curation. **Yanjie Li:** Supervision, Project administration.

## Declaration of competing interest

The authors declare that they have no known competing financial interests or personal relationships that could have appeared to influence the work reported in this paper.

## Appendix Supplementary materials

Image, application 1

Image, application 2
