# Supplementary material for: The role of lipids in mediating the effects of immune cells on Alzheimer’s disease risk: A network Mendelian randomization study
Source: J Prev Alzheimers Dis. 2026 Feb 20;13(4):100509. doi: 10.1016/j.tjpad.2026.100509 (PMC12934311; doi:10.1016/j.tjpad.2026.100509)

**MR Analysis of Immune Cells on AD**

**The data visualizations of Radial MR**

Naive CD4+ %T cell


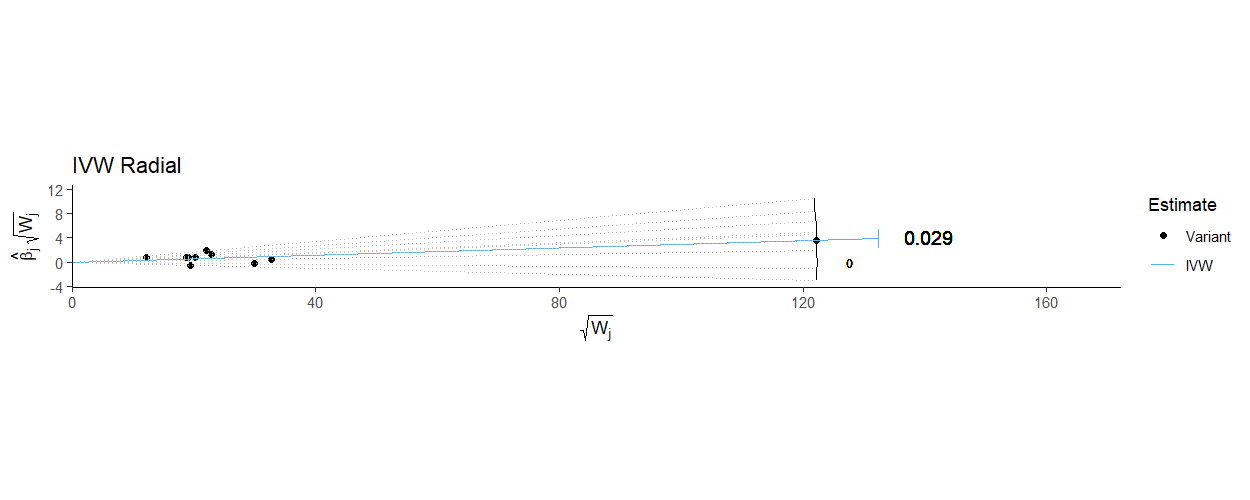


CD33 on CD14+ monocyte


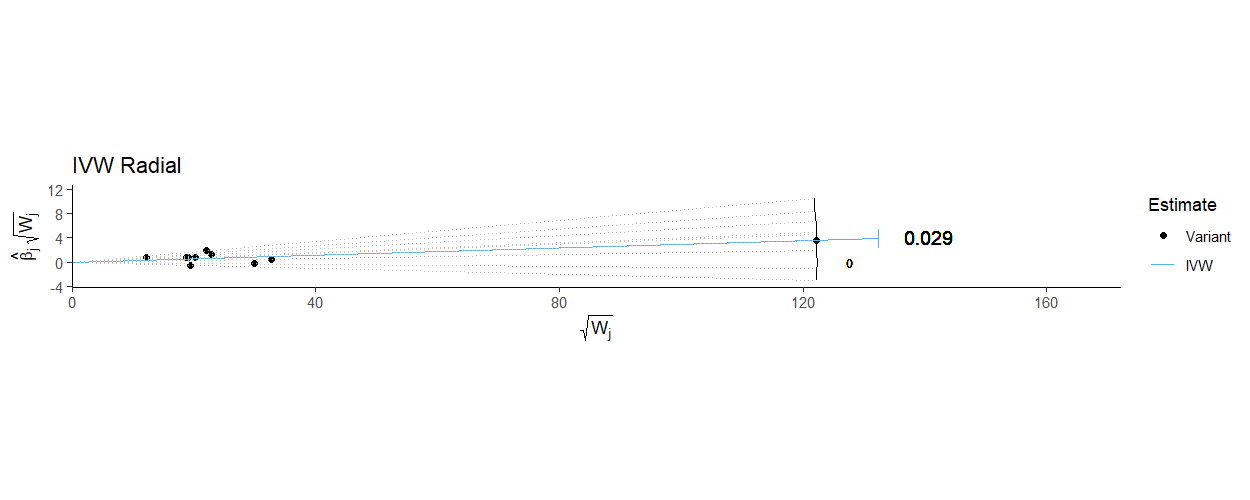


CD33 on CD33dim HLA DR+ CD11b+


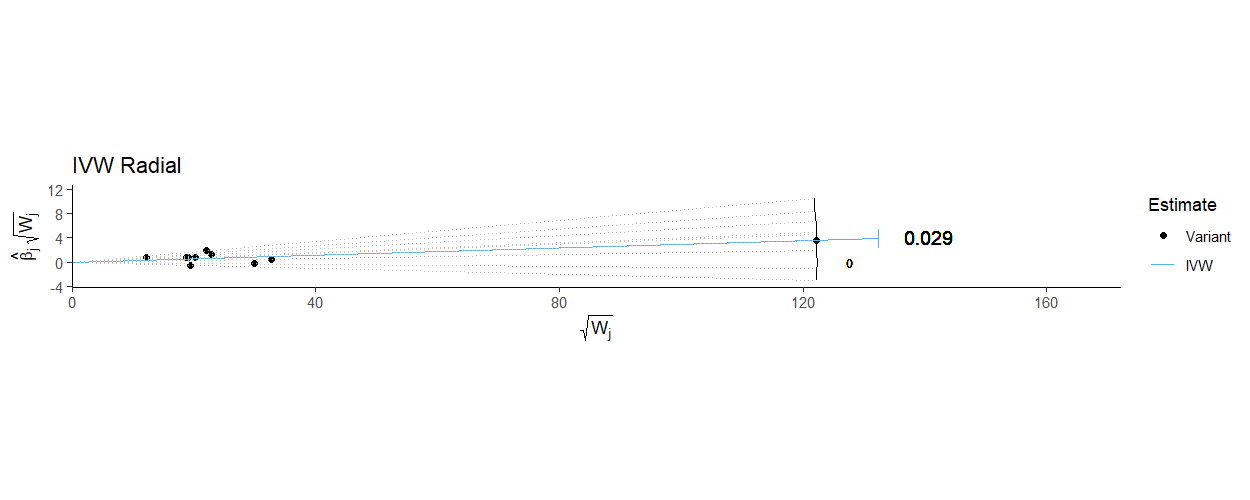


CD33 on CD33dim HLA DR+ CD11b-


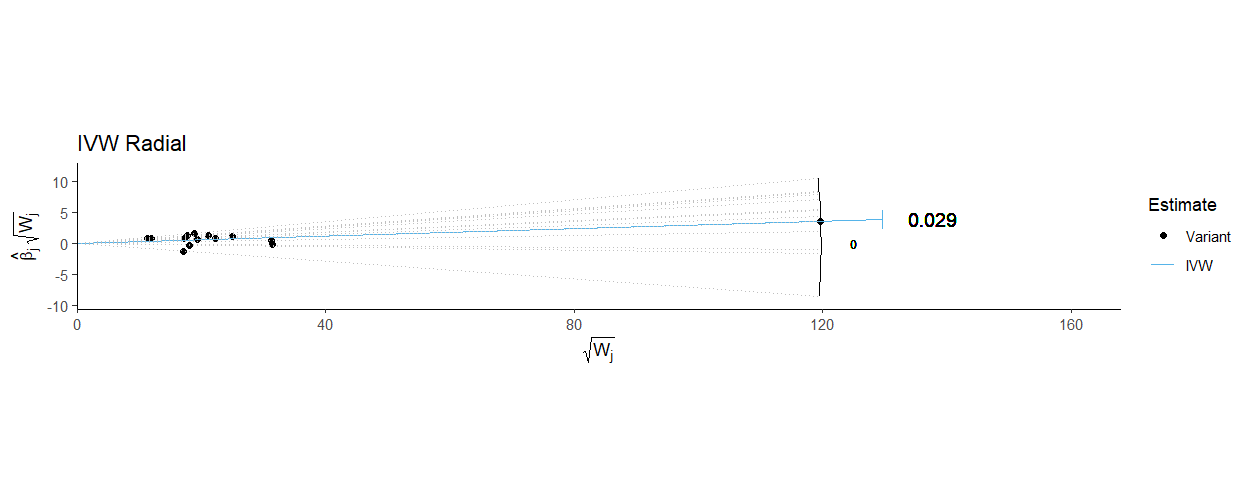


CD45 on CD33- HLA DR+


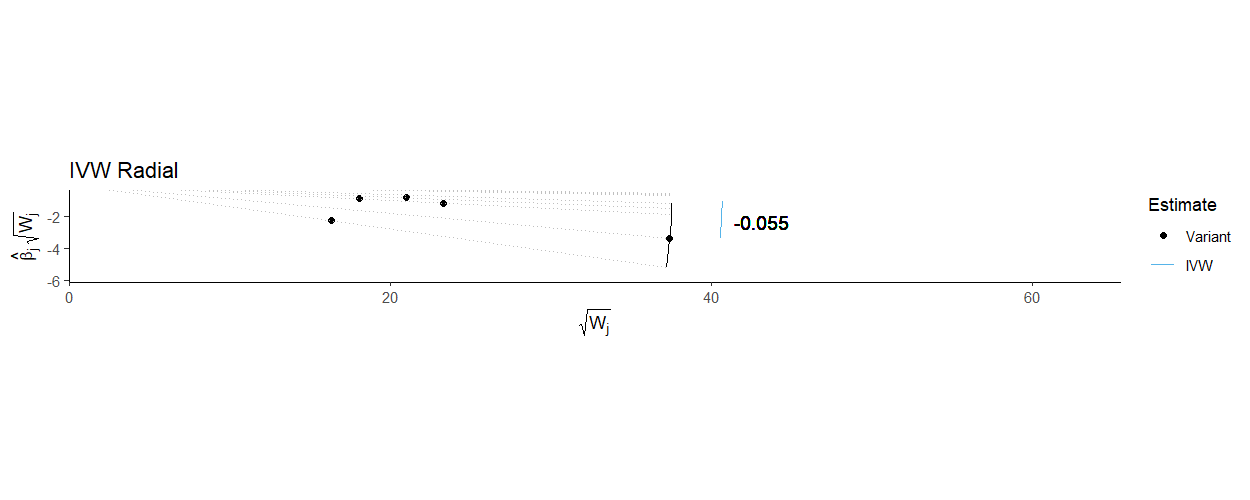


CD45 on CD33br HLA DR+


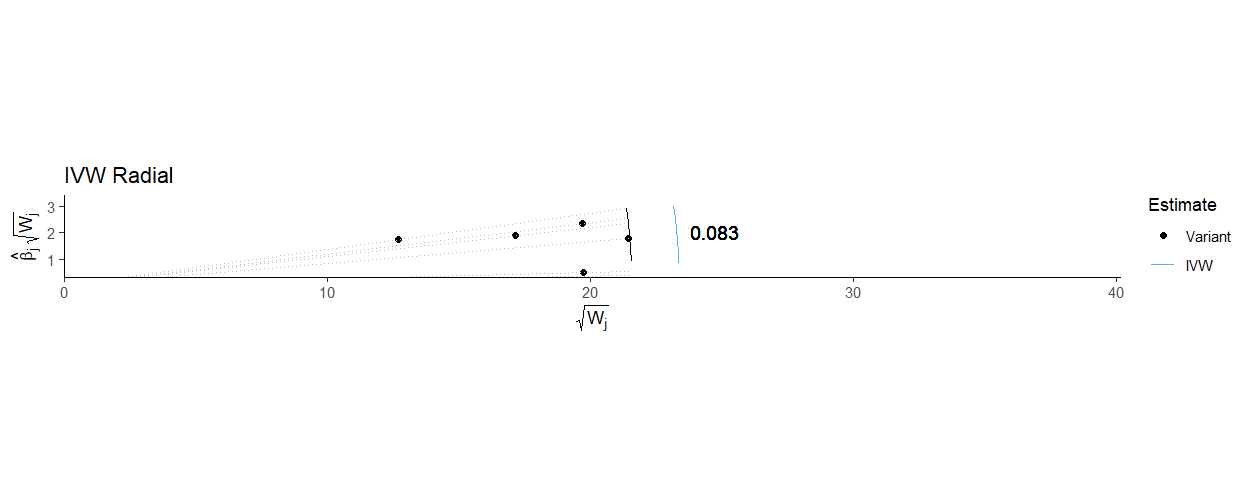
**The data visualizations of leave-one-out analysis**

Naive CD4+ %T cell


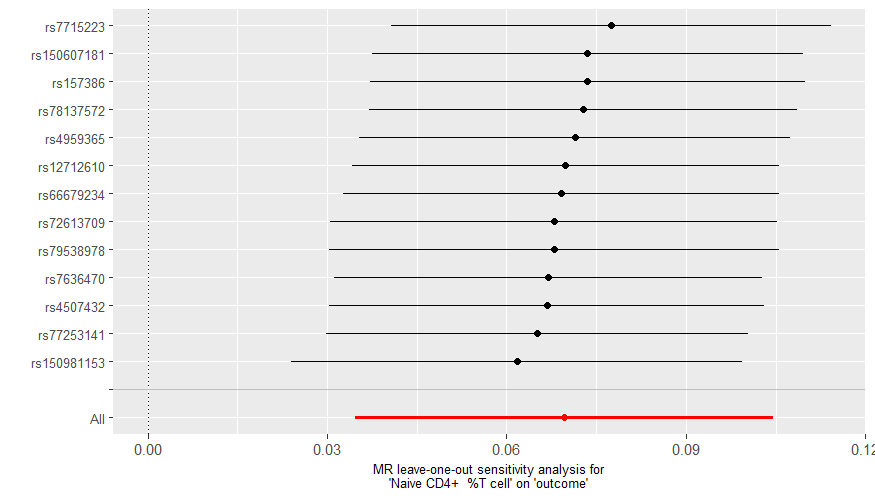


CD33 on CD14+ monocyte


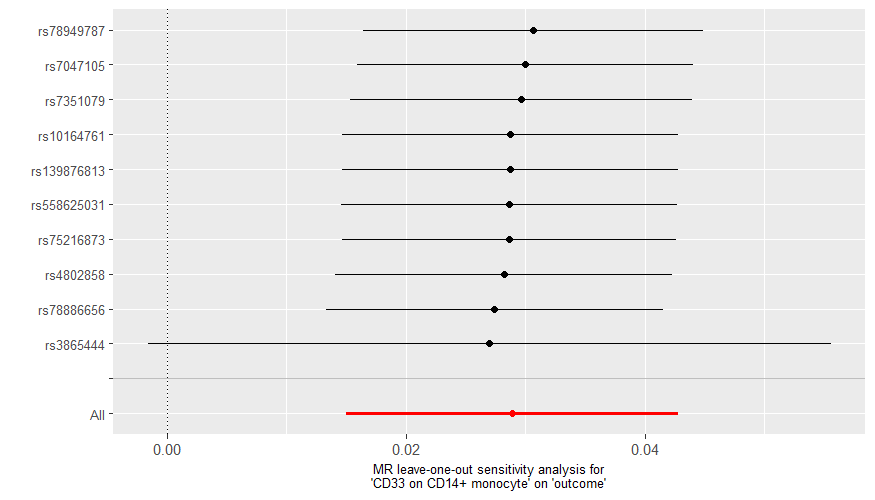


CD33 on CD33dim HLA DR+ CD11b+


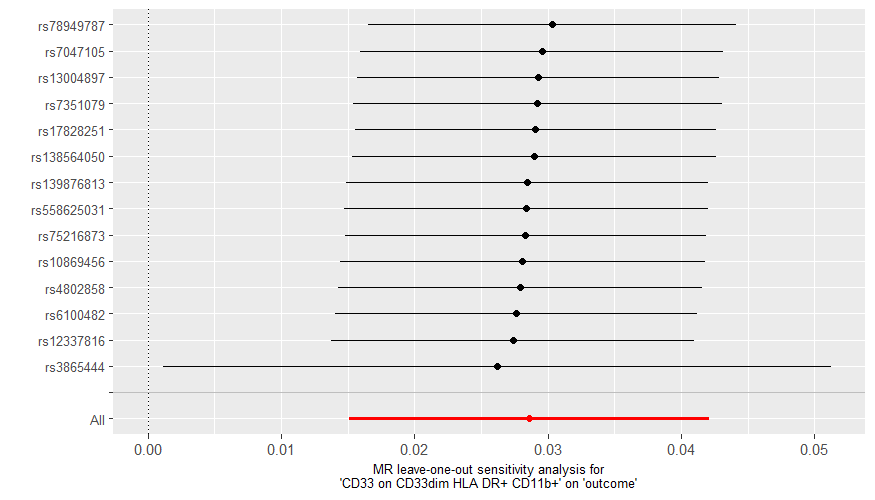


CD33 on CD33dim HLA DR+ CD11b-


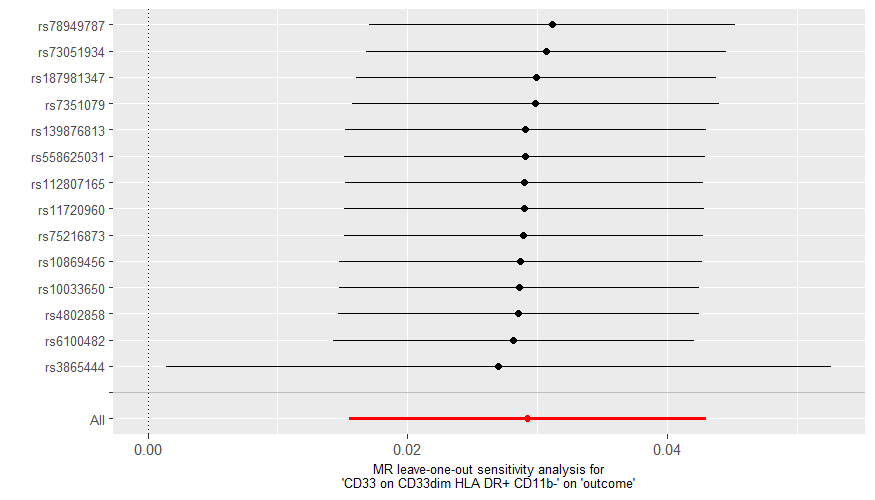


CD45 on CD33- HLA DR+


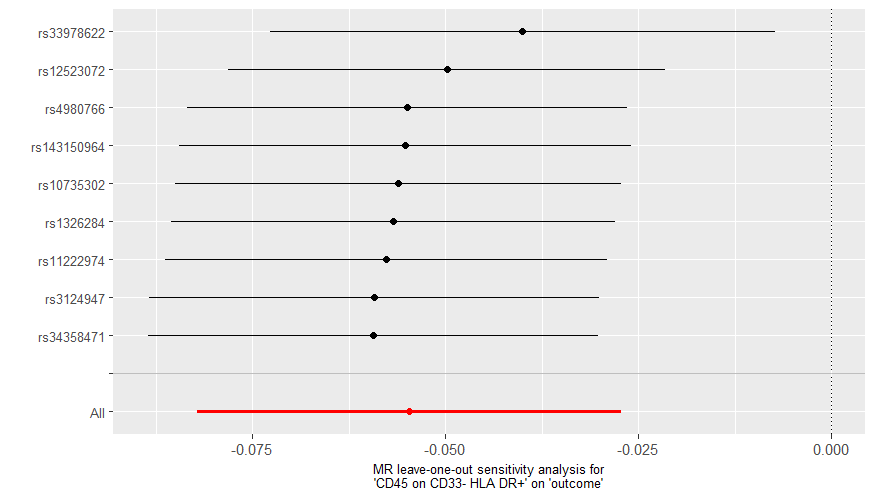


CD45 on CD33br HLA DR+


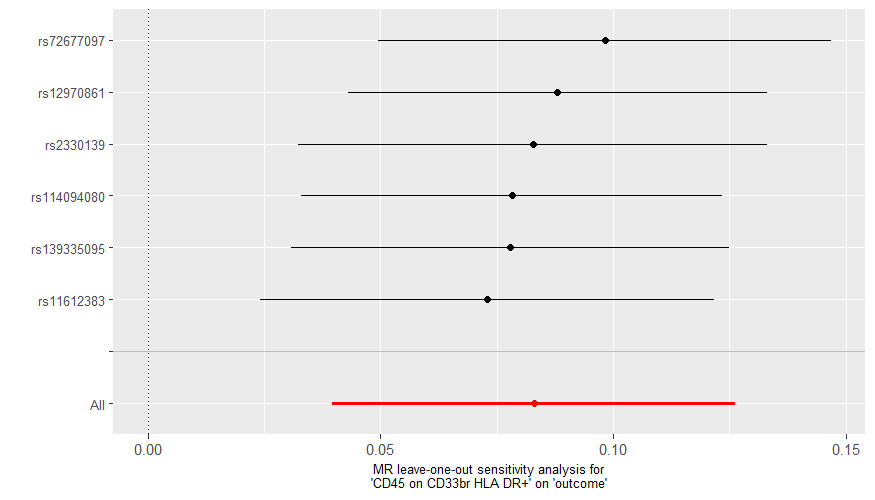
**The data visualizations of leave-one-chromosome-out analysis**

Naive CD4+ %T cell


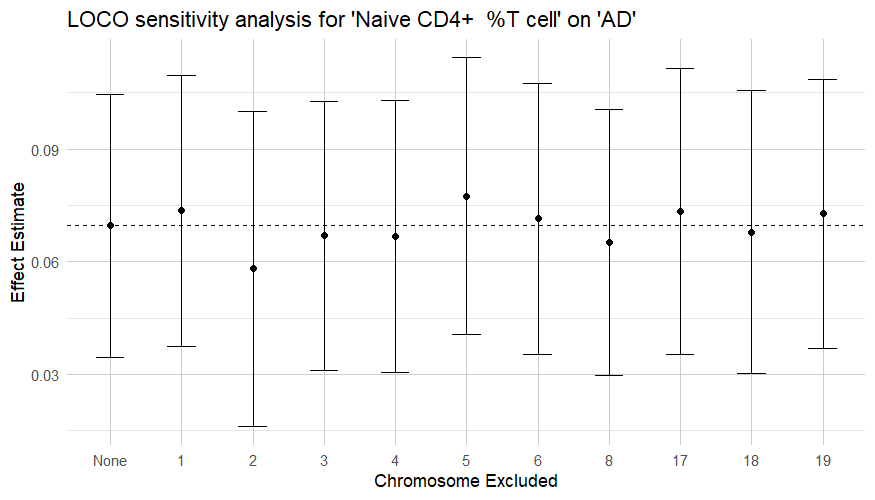


CD33 on CD14+ monocyte


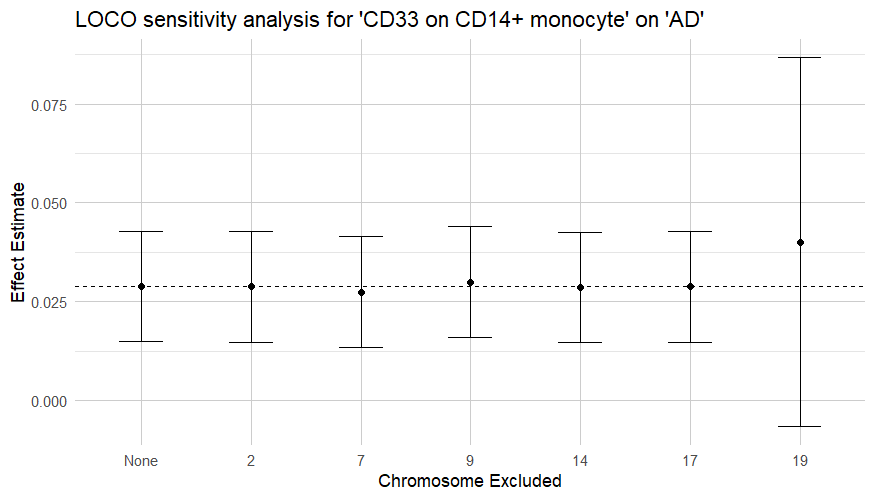


CD33 on CD33dim HLA DR+ CD11b+


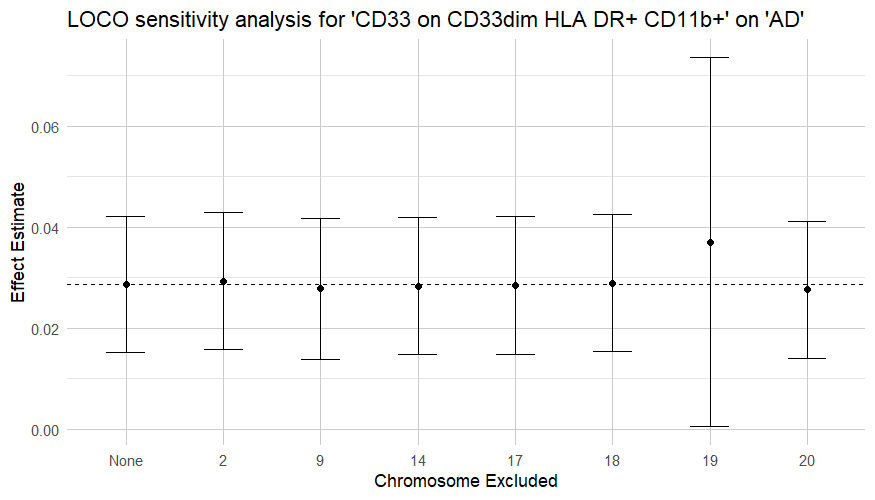


CD33 on CD33dim HLA DR+ CD11b-


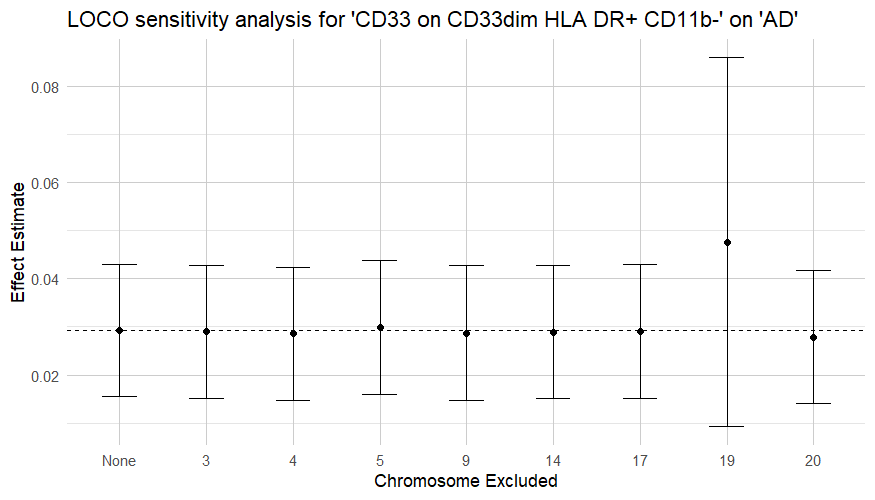


CD45 on CD33- HLA DR+


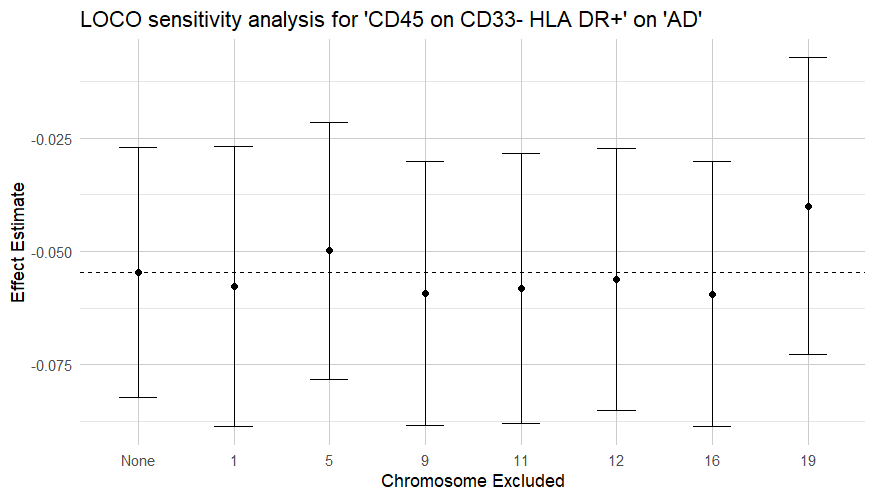


CD45 on CD33br HLA DR+


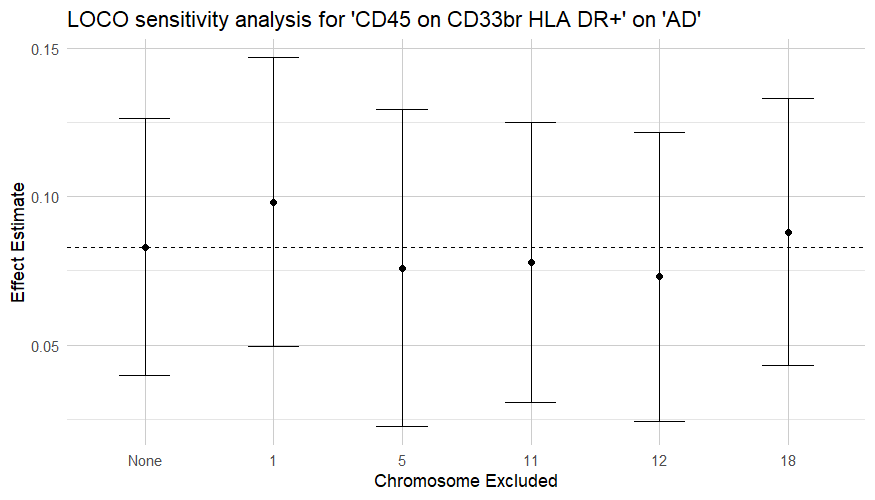


**The scatter plots**

Naive CD4+ %T cell


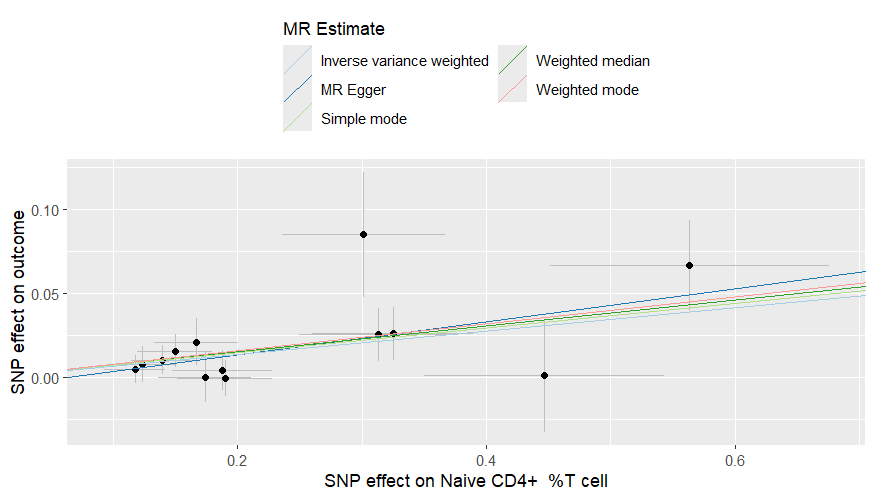


CD33 on CD14+ monocyte


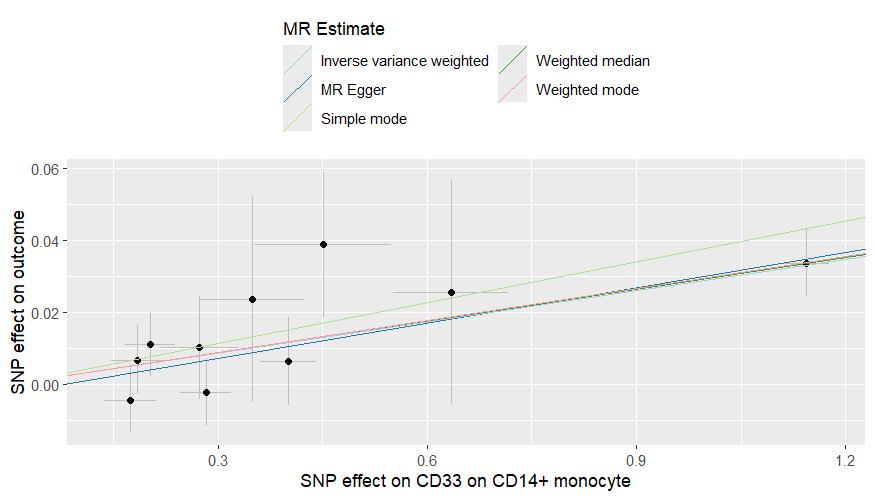


CD33 on CD33dim HLA DR+ CD11b+


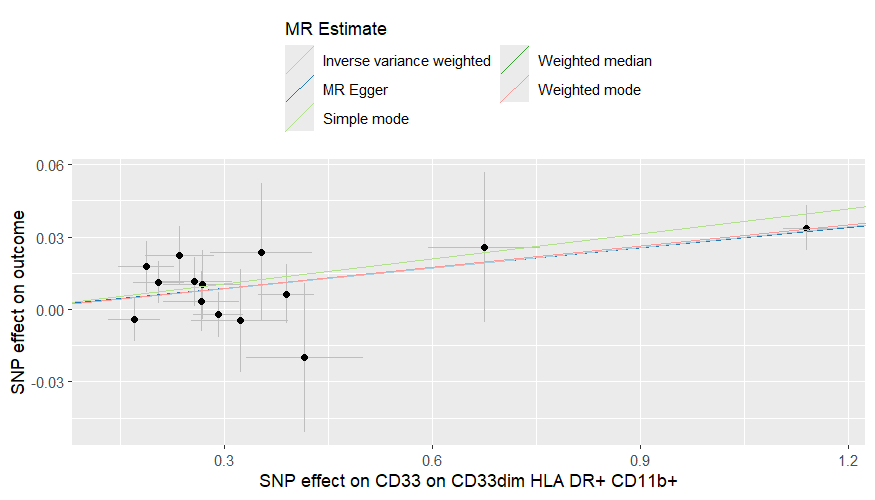


CD33 on CD33dim HLA DR+ CD11b-


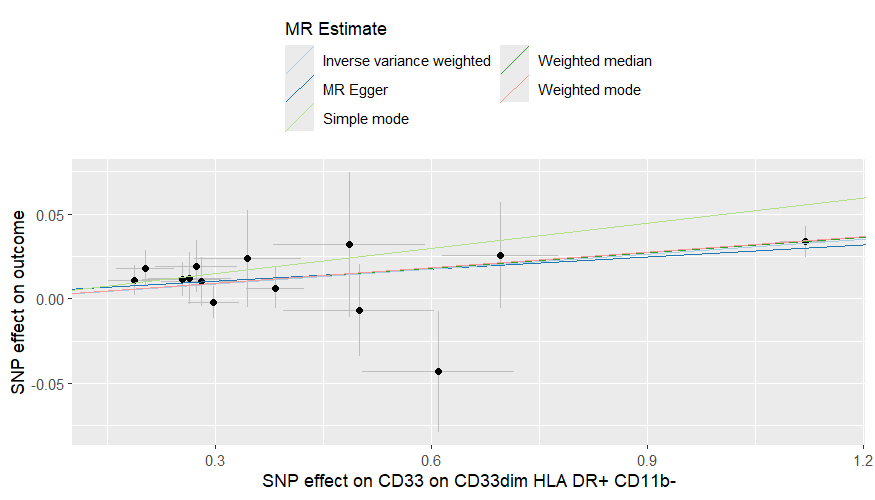


CD45 on CD33- HLA DR+


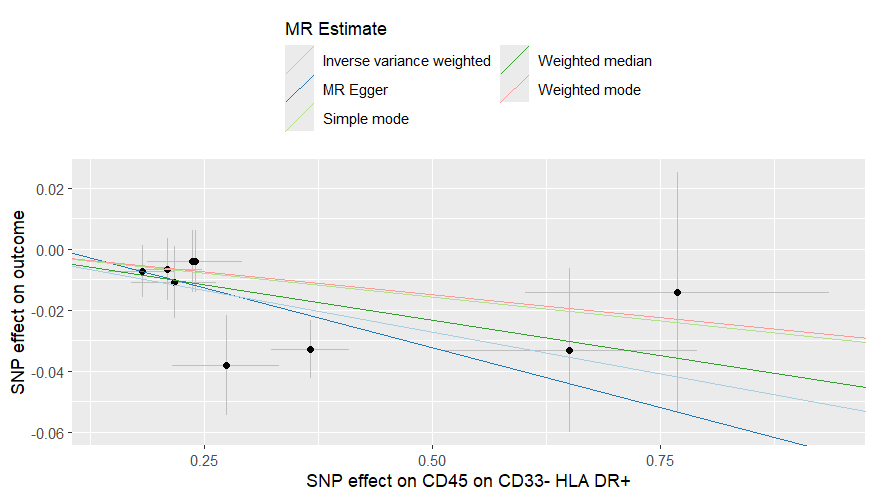


CD45 on CD33br HLA DR+


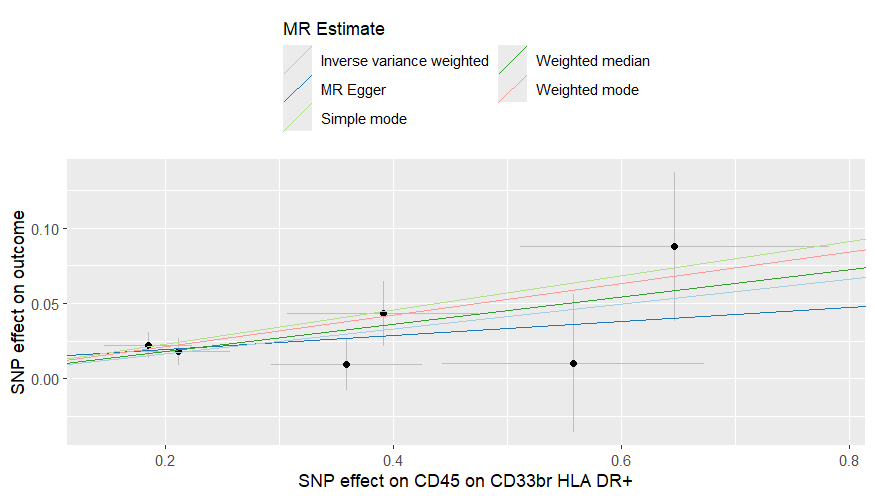

Supplement: Supplementary file 1 [file mmc1.docx]
